# Case Report: Pure Red Cell Aplasia Caused by Refractory Parvovirus B19 Infection After Pancreas Transplantation Alone

**DOI:** 10.3389/fmed.2022.849783

**Published:** 2022-03-16

**Authors:** Jørn Petter Lindahl, Regine Barlinn, Ingerid Weum Abrahamsen, Signe Spetalen, Karsten Midtvedt, Trond Jenssen

**Affiliations:** ^1^Department of Transplantation Medicine, Oslo University Hospital, Rikshospitalet, Oslo, Norway; ^2^Department of Microbiology, Oslo University Hospital, Rikshospitalet, Oslo, Norway; ^3^Department of Hematology, Oslo University Hospital, Rikshospitalet, Oslo, Norway; ^4^Department of Pathology, Oslo University Hospital, Radiumhospitalet, Oslo, Norway; ^5^Institute of Clinical Medicine, University of Oslo, Oslo, Norway

**Keywords:** type 1 diabetes (T1D), pancreas transplant alone, immunosuppression, anemia, parvovirus B19, pure red cell aplasia (PRCA), case report

## Abstract

A multidisciplinary team of doctors is in charge or is involved in the follow-up of patients who undergo solid organ transplantation (SOT). Immunosuppressive drugs are required after SOT, some potential unwanted side effects can be difficult to detect, and physicians must be aware of potential pitfalls. We report a case of a recipient with brittle type 1 diabetes who experienced severe and refractory anemia after pancreas transplantation alone (PTA). Despite a broad diagnostic approach for anemia, the diagnosis was delayed. The patient had normocytic normochromic anemia with severe reticulocytopenia and marked reduction or absence of erythroid precursors in the bone marrow, compatible with pure red cell aplasia (PRCA). Analyses of serological parvovirus B19 anti-IgM and anti-IgG antibodies, including PCR, were initially inconclusive/negative. The diagnosis of parvovirus B19 infection was confirmed after bone marrow biopsy with immunohistochemical staining for parvovirus B19. A retrospective analysis revealed an early post-transplant primary parvovirus B19 infection. The patient was successfully treated with intravenous immunoglobulin (IVIg) therapy. There is a risk of diagnostic delay for the less common types of anemia following SOT. Parvovirus B19 infection-associated PRCA is curable in SOT recipients and should be actively considered in patients with persistent anemia and low reticulocytes.

## Introduction

Anemia, defined as hemoglobin <12 g/dL in women and <13 g/dL in men, is a frequent problem following solid organ transplantation (SOT), which may be caused by per- and postoperative blood loss, iron and/or erythropoietin deficiency, infection prophylaxis medication, immunosuppressive drugs, use of angiotensin-converting enzyme inhibitors or angiotensin-receptor blockers, or viral infections. There are usually several differential diagnoses to consider, and most often more than one cause must be considered. Thus, there is a risk of diagnostic delay for the less common types of anemia.

Among the viral causes of anemia following SOT, parvovirus B19 infection can cause severe acute and chronic anemia in immunocompromised patients (1). Parvovirus B19 is a small capsid, approximately 25 nm in diameter, containing a genome consisting of single-stranded DNA (2, 3). Parvovirus B19 infection is common in childhood, and more than half of adolescents have parvovirus B19 specific antibodies (4, 5). Respiratory droplets transmit parvovirus B19 (6, 7). Up to half of adults experience asymptomatic infections. If symptomatic, flu-like symptoms are common during the viremia phase of the infection, followed by rashes and/or joint pain. Children are more likely to develop rashes, and adults tend to experience joint pain. Parvovirus B19 can also be transmitted through the blood or blood products and transplanted organs that contain the virus (8).

Parvovirus B19 infects and replicates in erythroid progenitor cells in the bone marrow, and viral replication results in apoptosis of infected cells (7). Therefore, a decrease or even absence of reticulocytes is a hallmark of parvovirus B19 infection, even in immunocompetent individuals. In some cases, transient anemia and other cytopenias are observed. Parvovirus B19 infection can cause transient aplastic crisis in individuals with underlying hemolytic disorders such as hereditary spherocytosis and sickle cell disease, fetal loss and hydrops fetalis in pregnancy, and pure red cell aplasia (PRCA) in immunocompromised patients (1).

Pure red cell aplasia, a less common cause of anemia, is limited to the red cell lineage and is characterized by normocytic normochromic anemia with severe reticulocytopenia and marked reduction or absence of erythroid precursors in the bone marrow (9). Acquired PRCA may be caused by lymphoproliferative disorders, immunologic/autoimmune disorders, drugs, viral infections (most commonly parvovirus B19 infection), thymoma, and other cancers (9). Patients treated with immunosuppressive drugs are more likely to develop parvovirus B19 infection-related PRCA due to sustained viremia and suppression of erythropoiesis.

The treatment of parvovirus B19 infection-related complications in SOT recipients involves blood transfusion, reduction in immunosuppression, and intravenous immunoglobulin (IVIg) therapy (1, 10–14).

This case report, will review refractory parvovirus B19-induced PRCA in a patient who received a single pancreas graft.

## Case Description

A 39-year-old man was admitted to our national transplant hospital ward with fever, myalgia, and neutropenia.

Due to brittle type 1 diabetes mellitus he had 6 months earlier undergone successful transplantation with a single pancreas graft obtained from a deceased donor. Donor-recipient ABO blood group match was O-O, HLA-match was 3-1 ([HLA-A 1 + HLA-B 2]-[HLA-DR 1]), and cytomegalovirus (CMV) serologic status was donor IgG antibody-positive to recipient IgG antibody-negative. At the time of transplantation, the recipient was positive for Epstein-Barr virus (EBV) and varicella zoster IgG antibodies. Pancreas transplantation was performed with exocrine drainage through anastomosis with the recipient’s duodenum.

Postoperatively, there was immediate pancreatic transplant function without the need for exogenous insulin. Immunosuppression consisted of induction therapy with a single dose of 250 mg methylprednisolone intravenously at the time of transplantation and T-cell monitored administration of thymoglobulin intravenously for the 10 first days post-transplant, with an accumulated dose of 500 mg (200, 150, and 150 mg on days 0, 3, and 8 post-transplant, respectively) (15). Maintenance immunosuppression included tacrolimus, which was adjusted to maintain trough levels between 10 and 12 ng/mL for the first 8 weeks, and thereafter 6–10 ng/mL, mycophenolate mofetil 1 g administered orally twice daily, and standard prednisone tapered to 5 mg once daily after 6 months. In addition, he received standard treatment with sulfamethoxazole 400 mg/trimethoprim 80 mg as Pneumocystis jiroveci prophylaxis, and due to CMV-seronegative recipient of allograft from a CMV-seropositive donor, he also received pre-emptive valganciklovir treatment, both planned for 6 months.

The recipient received prophylactic valaciklovir for 1 week from post-operative day eight due to assumed varicella exposure from an adolescent female friend. The patient was discharged on the twelfth post-operative day with excellent pancreatic allograft function. Laboratory tests at discharge revealed a hemoglobin of 9.2 g/dL (13.5 g/dL at the time of transplantation), total leukocyte count of 8.0 × 10^9^/L, and platelet count of 324 × 10^9^/L.

On the twenty-sixth post-operative day, he received a blood transfusion (two units of SAGMAN erythrocytes) for a decline in hemoglobin concentration (from 9.4 to 7.1 g/dL). The decrease in hemoglobin level was thought to be caused by an older hematoma in relation to the pancreas transplant, and due to stabilization of hemoglobin after blood transfusion, no surgery or other interventions were considered necessary.

During the first 6 months after engraftment, the mycophenolate mofetil dose was either reduced or paused several times owing to varying degrees of leukopenia and neutropenia. Sulfamethoxazole/trimethoprim and valganciklovir were discontinued for the same reasons.

On post-transplant day one hundred seventy-nine, the patient presented with a 2–3 days history of low-grade fever, leukopenia, neutropenia, and myalgia. On presentation, the patient had a hemoglobin of 11.9 g/dL, total leukocyte count of 1.3 × 10^9^/L, neutrophil count of 0.8 × 10^9^/L, platelet count of 164 × 10^9^/L, and C-reactive protein level of 2.7 mg/L. His blood glucose and C-peptide levels were within normal limits. Maintenance medication at admission, in addition to immunosuppressive drugs as mentioned above, consisted of once-daily acetylsalicylic acid 75 mg, twice-daily pantoprazole 40 mg, and once-daily dalteparin 5000 IU administered subcutaneously. Sulfamethoxazole/trimethoprim and valganciklovir had already been discontinued at admission due to leukopenia and neutropenia, and finally stopped according to a protocol that states prophylaxis for 6 months.

The mycophenolate mofetil dose was discontinued at admission, and after 1 week reintroduced gradually over the next 2 weeks to 750 mg twice daily. The CMV serological status (IgM and IgG antibodies) was negative, and whole blood PCR for CMV showed no detectable CMV DNA sequence in view of a presumed CMV primary disease. EBV serological and PCR results were also negative. Granulocyte colony-stimulating factor (G-CSF) was not administered. Neutrophils were at its lowest 0.6 × 10^9^/L. The bacterial cultures were sterile and the standard respiratory viral panel test results were negative. Cefotaxim was initially administered intravenously for neutropenic fever. After 5 days, his symptoms improved, and he was discharged with a hemoglobin of 10.4 g/dL, total leukocyte count of 1.6 × 10^9^/L, neutrophils of 0.8 × 10^9^/L, and a platelet count of 186 × 10^9^/L. Within 6 days of discharge, the patient’s leukocyte and neutrophil counts increased to normal levels, and the complete blood count showed a hemoglobin of 10.9 g/dL, total leukocyte count of 3.9 × 10^9^/L, neutrophil count of 2.4 × 10^9^/L, and platelet count of 384 × 10^9^/L.

**FIGURE 1:**
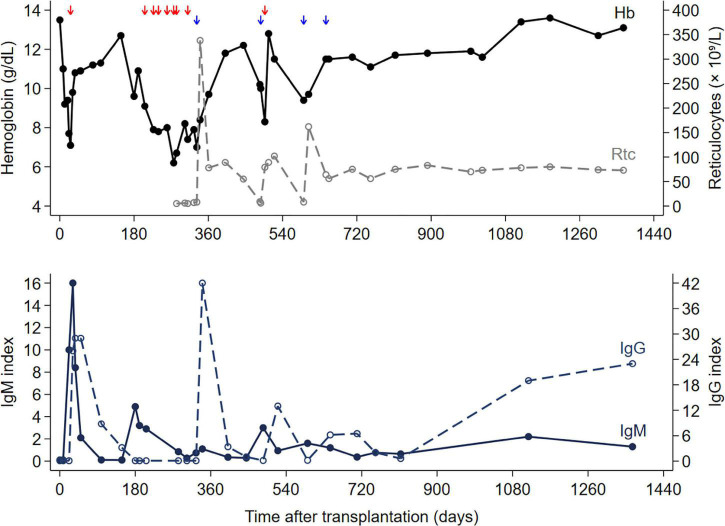
Hemoglobin level, reticulocyte count, IgM and IgG indexes, and time of blood transfusions (red arrow), and IVIg therapy (blue arrow) after transplantation.

During the following month after discharge and forward, the patient repeatedly presented with anemia (normocytic normochromic), and he received blood transfusions altogether eight times (at least two SAGMAN erythrocytes per transfusion) between post-operative day two-hundred and six and three-hundred and ten (Figure 1). Evaluation for occult bleeding, including gastroscopy, and hemolysis parameters (haptoglobin, LDH, and bilirubin) were normal. Serum protein electrophoresis, iron status, and vitamin B_12_ and folate levels were also normal. The patient received darbepoetin alfa without any significant effects on anemia. His tacrolimus levels were within target range 6–10 ng/mL, and therapeutic drug monitoring of mycophenolate mofetil (750 mg twice daily) revealed an area under the concentration time curve of 56.6 mg × h/L (16).

On post-operative day two hundred eighty-three, results of laboratory tests revealed a reticulocyte count of <5% (Figure 1), which was compatible with pure red cell anemia. The immunosuppressive regimen was switched from a tacrolimus/mycophenolate mofetil to a cyclosporine/everolimus-based regimen because of a suspected association between immunosuppressive drugs and PRCA (9, 17). Darbepoetin alfa was discontinued for the same reason (18, 19). The patient tested negative for anti-erythropoietin antibodies. Furthermore, parvovirus B19 specific IgG antibodies were negative, whereas IgM antibodies were reported as gray zones (Table 1). The parvovirus B19 PCR results were inconclusive (Table 1). A CT scan of the chest was performed for possible thymoma-associated PRCA, but there was no sign of thymoma (20).

**TABLE 1:** Parvovirus B19 serologic status and parvovirus B19 PCR of the recipient and donor at different times after transplantation.

Variable	Parvovirus B19 specific analyses			
	IgG index^a^	IgM index^a^	PCR^b^	PCR 1:100^b^
Donor				
Day 0	*Positive* (16)*^c^*	Negative (0.1)^c^	Negative^c^	
Recipient				
Day 0	Negative (<0.1)^c^	Negative (<0.1)^c^	Negative^c^	
Day 8	Negative (<0.1)^c^	Negative (<0.1)^c^	Negative^c^	
Day 22	Negative (<0.1)^c^	*Positive (10.0)^c^*	Inconclusive^c^	*Positive* ^c^
Day 31	*Positive (26.0)^c^*	*Positive (16.0)^c^*	*Positive* ^c^	
Day 37	*Positive (29.0)^c^*	*Positive (8.4)^c^*	*Positive* ^c^	
Day 50	*Positive (29.0)^c^*	*Positive (2.1)^c^*	*Positive* ^c^	Negative^c^
Day 99	*Positive (8.8)^c^*	Negative (0.1)^c^	*Positive* ^c^	Negative^c^
Day 148	*Positive (3.2)^c^*	Negative (<0.1)^c^	*Positive* ^c^	Negative^c^
Day 180	Negative (<0.1)^c^	*Positive (4.9)^c^*	Inconclusive^c^	*Positive* ^c^
Day 190	Negative (<0.1)^c^	*Positive (3.2)^c^*	Inconclusive^c^	*Positive* ^c^
Day 206	Negative (<0.1)^c^	*Positive(2.9)^c^*	Inconclusive^c^	*Positive* ^c^
Day 283	Negative (<0.1)	Equivocal (0.9)	Inconclusive	*Positive* ^c^
Day 303	Negative (<0.1)	Negative (0.3)	Inconclusive	
Day 325	Negative (<0.1)	Equivocal (0.7)	Inconclusive	*Positive* ^d^
Day 340	*Positive (42.0)*	Equivocal (1.1)^c^	*Positive*	
Day 401	*Positive (3.4)*	Negative (0.4)^c^	*Positive*	
Day 445	Equivocal (1.0)	Negative (0.3)^c^	*Positive*	
Day 485	Negative (0.2)	*Positive (3.0)*	Inconclusive	*Positive*
Day 520	*Positive (13.0)*	Equivocal (0.9)^c^	*Positive*	
Day 591	Negative (0.2)	*Weak positive (1.6)*	Inconclusive	*Positive*
Day 645	*Positive (6.2)*	Equivocal (1.2)^c^	*Positive*	
Day 709	*Positive (6.5)*	Negative (0.4)	*Positive*	
Day 753	*Weak positive (2.0)*	Equivocal (0.8)^c^	*Positive*	
Day 813	Negative (0.6)	Equivocal (0.7)^c^	*Positive*	
Day 1118	*Positive (19.0)*	*Positive (2.2)*	*Positive*	
Day 1366	*Positive (23.0)*	Equivocal (1.3)	*Positive*	

*^a^LIAISON^®^ Biotrin Parvovirus B19 IgG, IgM (DiaSorin).*

*^b^In-house accredited PCR assay with primers and probes, which target the VP2 gene of the Parvovirus B19, performed undiluted and diluted 1:100.*

*^c^Retrospectively analyzed.*

*^d^PCR confirmed positive using another method and laboratory. Inconclusive results, such as negative PCR and internal control, for example, inhibitory factors may affect the results.*

**FIGURE 2:**
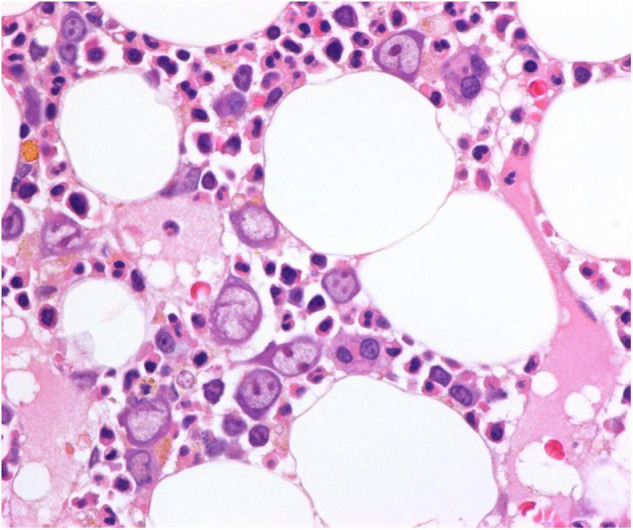
Bone marrow biopsy at time of diagnosis, hematoxylin and eosin (H&E) stain, original magnification 600×. Giant proerythroblasts are present, and some of them contain prominent eosinophilic nuclear inclusion bodies. Other stages of erythroid maturation are markedly reduced.

**FIGURE 3:**
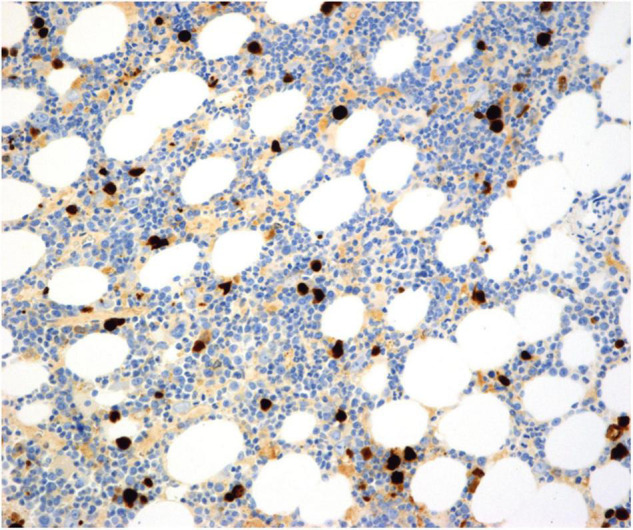
Bone marrow biopsy at time of diagnosis. An immunohistochemical stain for parvovirus B19 highlights the infected cells. Parvovirus B19, clone R92F6, Abcam production number ab64295. Original magnification 200×.

A bone marrow aspirate showed morphological changes of pure red cell aplasia, presence of giant pro-erythroblasts, and absence of nucleated red cells. Parvovirus B19 infection was still a possible differential diagnosis, but the serologic status was negative or inconclusive, and parvovirus B19 PCR was inconclusive (Table 1). Due to persistent transfusion refractory anemia and no effect on the low hemoglobin levels after a change in immunosuppressive therapy, a bone marrow biopsy was performed. Bone marrow biopsy revealed substantial erythroid hypoplasia with a marked left shift and giant proerythroblasts, some with intranuclear inclusions (Figure 2). Immunohistochemical staining for parvovirus B19 was positive and compatible with parvovirus B19 infection-associated PRCA (Figure 3). Eventually, parvovirus B19 was also demonstrated by PCR in the blood on reexamination and evaluation by performing a dilution series (Table 1).

The patient responded well to treatment with 30 g (0.4 g/kg/day) intravenous immunoglobulin (IVIg) for six consecutive days. His reticulocyte levels normalized at the end of the IVIg therapy period, and hemoglobin levels gradually increased during the next 4 weeks to 10–11 g/dL (Figure 1).

However, the patient experienced two relapses (Figure 1), 4 and 8 months, respectively, after the initial IVIg treatment. The patient was treated with IVIg on both occasions. At the first relapse, IVIg treatment had a transient effect; however, at the second relapse, there was a sustained effect. A follow-up bone marrow biopsy was performed approximately 1 year (on post-transplant day seven hundred nine) after the first round of IVIg therapy due to lack of seroconversion as well as sustained parvovirus B19 viremia, at which time the patient had experienced both relapses. Bone marrow biopsy revealed normal maturation of all three cell lineages, including the erythroid lineage. Immunohistochemical staining for parvovirus B19 yielded negative results. At the time of the last relapse, the patient was treated with an extra round of IVIg, a type of consolidation therapy, to prevent further relapse (Figure 1). Eventually, the patient seroconverted with elevated titers of IgG specific for parvovirus B19, controlled for possible IVIg-interaction, and the hemoglobin level has been stable for the last 2 years at 11–13 g/dl, likewise, the reticulocyte count was in the normal range.

To assess the possible donor parvovirus B19 transmission, the donor serum was tested for parvovirus B19 antibody titers and showed that the donor was positive for parvovirus B19 specific IgG antibodies, but negative for IgM antibodies and parvovirus B19 PCR.

## Discussion

To the best of our knowledge, this is the second report of severe anemia due to refractory parvovirus B19 infection-associated PRCA after pancreas transplantation alone (PTA) (21). In contrast to the first report, our patient experienced two relapses after the initial IVIg treatment.

Parvovirus B19 infection following SOT may be due to blood transfusions or may be acquired through primary infection from exposure. Donor transmission from transplanted organs is unlikely. Although parvovirus B19 DNA regularly persists in different tissues, reactivation and replication are unlikely in this virus type. Studies have shown that the risk of acquiring a parvovirus B19 infection is greater during the 1st year post-engraftment and can be related to the use of red blood cell transfusions (22, 23).

The patient in this case report was negative for parvovirus B19 specific IgG and IgM antibodies and parvovirus B19 PCR at the time of transplantation, which ruled out persistent parvovirus B19 infection prior to the transplantation. We do not routinely screen for parvovirus B19 serologic status before transplantation. In addition, the patient first received blood transfusion after parvovirus B19 specific IgM antibodies, and PCR was documented as positive postoperatively (by retrospective analyses), which precludes blood transfusion-transmitted parvovirus B19 infection.

The deceased donor had previously undergone parvovirus B19 infection (presence of IgG antibodies), but had no sign of an acute infection (IgM and parvovirus B19 PCR negative). Therefore, the transmission of parvovirus B19 from the donor is less likely. A female friend who visited the recipient on postoperative day seven developed a skin rash the day after being in the ward. It was assumed that the visitor had a varicella zoster virus infection. In the retrospective analysis, the recipient developed parvovirus B19 specific IgM antibodies and was parvovirus B19 PCR positive 2 weeks after this exposure (Table 1). Based on these data, it seems more likely that the visitor had an acute parvovirus B19 infection and subsequently infected the patient.

There are several pitfalls and potential delays in diagnosing parvovirus B19 infection after SOT (24). The clinical presentation can vary from asymptomatic to life-threatening. Anemia is also a common clinical problem after transplantation, and at first sight, there are often other more likely explanations for anemia. Serologic parvovirus B19 IgM (or IgG) testing is not always useful for the diagnosis of acute infection in immunocompromised patients because seroconversion may be delayed or not occur. False-negative serology due to virus-antibody complexes is not uncommon and is more likely in acutely infected patients with high-level viremia (25). Table 1 presents the results. However, reticulocyte evaluation is a simple test that can be helpful.

In this case, the recipient’s diagnosis of parvovirus B19 infection was delayed. It was first captured by immunohistochemistry analysis of a bone marrow biopsy, despite two PCR DNA examinations of blood samples and serologic analyses. Early tests were inconclusive because of methodological problems caused by huge virus titers and lack of dilution series. In addition, in retrospective analyses, the patient had mounted an early humoral immune response, being first IgM positive, followed by IgG positive and parvovirus B19 PCR positivity at the time of early post-transplant parvovirus B19 exposure. Therefore, the patient first became sick already 2 weeks after exposure (at the time of first transfusion), and he again developed transfusion requiring anemia nearly 6 months later (and thereafter he was first diagnosed with parvovirus B19 infection), with the same high-level parvovirus B19-titer/DNAemia (Table 1). At that time, the serologic status was also negative or inconclusive. One explanation for this could be the high viral load and the formation of immune complexes (25). In our patient, delay in the diagnosis of parvovirus B19 infection resulted in additional diagnostic workup, including endoscopy and radiological imaging. In addition, the patient received erythropoietin therapy and multiple blood transfusions.

There are reports of the treatment of parvovirus B19-related PRCA with intravenous immunoglobulin (IVIg) (1, 11–14). PRCA, secondary to parvovirus B19 infection, is reported to be corrected after the first course of IVIg in 93% of patients, but approximately one-third relapse after a mean time of 4.3 months (14). Reduction of immunosuppression should be considered at the time of parvovirus B19 infection diagnosis (12). Relapse of reticulocytopenia and anemia has been reported in up to one-third of cases (12, 14). Currently, no specific antiviral drugs are available for the treatment of parvovirus B19 infection (26). Routine screening of donor and recipient serologic status for parvovirus B19 is not recommended but may be performed retrospectively when warranted (12).

In conclusion, parvovirus B19 infection-associated PRCA is a severe but treatable condition in SOT recipients and should be actively considered in patients with persistent anemia and low reticulocytes.

## Data Availability Statement

The original contributions presented in the study are included in the article/supplementary material, further inquiries can be directed to the corresponding author.

## Ethics Statement

Ethical review and approval was not required for the study on human participants in accordance with the local legislation and institutional requirements. The patients/participants provided their written informed consent to participate in this study. Written informed consent was obtained from the individual(s) for the publication of any potentially identifiable images or data included in this article.

## Author Contributions

JPL is the guarantor of this work and, as such, had full access to all the data in the study and takes responsibility for the integrity of the data, and the accuracy of the data analysis. All authors contributed to the substantial contribution to the conception and design, acquisition of data, and/or analysis and interpretation of data, drafting the article and/or revising it critically for important intellectual content, and final approval of the version to be published.

## Conflict of Interest

The authors declare that the research was conducted in the absence of any commercial or financial relationships that could be construed as a potential conflict of interest.

## Publisher’s Note

All claims expressed in this article are solely those of the authors and do not necessarily represent those of their affiliated organizations, or those of the publisher, the editors and the reviewers. Any product that may be evaluated in this article, or claim that may be made by its manufacturer, is not guaranteed or endorsed by the publisher.

## Abbreviations

IVIg: intravenous immunoglobulin
PRCA: pure red cell aplasia
PTA: pancreas transplantation alone
SOT: solid organ transplantation.
